# Deep-sea ecosystems in the north-eastern Alboran Sea (western Mediterranean): quantifying assemblages and anthropogenic activity in the Seco de los Olivos Bank

**DOI:** 10.1007/s12526-025-01505-4

**Published:** 2025-03-22

**Authors:** Patricia Puerta, Rosa M. Cañedo-Apolaya, José L. Rueda, Carlos Dominguez-Carrió, Javier Urra, Covadonga Orejas

**Affiliations:** 1 Centro Oceanográfico de Baleares, Instituto Español de Oceanografía (IEO-CSIC), Palma, Spain; 2 Department of Biology, Marine Biology, Ghent University, Ghent, Belgium; 3 Centro Oceanográfico de Málaga, Instituto Español de Oceanografía (IEO-CSIC), Málaga, Spain; 4 Instituto de Investigação em Ciências do Mar – Okeanos, Universidade Dos Açores, Horta, Portugal; 5 IMAR Instituto Do Mar, Universidade Dos Açores, Horta, Portugal; 6 Centro Oceanográfico de Gijón, Instituto Español de Oceanografía (IEO-CSIC), Gijón, Spain

**Keywords:** Benthic communities, Vulnerable marine ecosystems, Remotely operated vehicle, Marine litter

## Abstract

Seafloor elevations (e.g., seamounts, banks, mounds) are essential underwater features for supporting and maintaining global marine biodiversity. Such geomorphological features might be of particular relevance to preserve biodiversity in the Mediterranean Sea, where a high number of anthropogenic impacts threaten deep-sea ecosystems. Using imagery from a remotely operated vehicle (ROV), deep-sea megabenthic and demersal fish assemblages were identified and quantified in some of the less studied areas of the Seco de los Olivos Bank (also known as Chella Bank), a seafloor elevation considered a hotspot of biodiversity in the Alboran Sea, Western Mediterranean. The 62 taxa identified were grouped into five well-defined benthic and demersal assemblages, mainly influenced by substrate type and typified by massive sponges, cold-water corals, sea pens, and ray-finned fishes. Nine taxa were identified as indicators of vulnerable marine ecosystems (VMEs) and/or endangered species. The heterogeneous distribution of substrate types plays a key role in assemblage composition, with hard substrates enhancing biodiversity at the local scale. A wide variety of indicators of anthropogenic activities were found, including bottom trawling marks, remains of fishing gears, and other types of marine litter, whose presence seemed to reduce the diversity of the observed assemblages. The results presented here improve the still scarce quantitative assessments of deep-sea benthic ecosystems to implement effective management measures in the framework of the main European policies (e.g., Habitats Directive, Marine Strategy Framework Directive) to reach 2030 conservation goals.

**Supplementary Information:**

The online version contains supplementary material available at 10.1007/s12526-025-01505-4.

## Introduction

Seamounts and other seafloor elevations are considered essential ecosystems worldwide for supporting and maintaining marine biodiversity (Clark et al. 2010b; Rowden et al. 2010; Würtz and Rovere 2015; Du et al. 2016; Watling and Auster 2017). They provide heterogeneous habitats and harbor rich and diverse biological communities (Clark et al. 2010a; McClain and Lundsten 2015; Rogers 2018; Victorero et al. 2018; Ramiro-Sánchez et al. 2019), including many vulnerable and threatened species (Davies et al. 2015; Ramos et al. 2016; Watling and Auster 2017; D’Onghia 2019). Nevertheless, these ecosystems have historically been exploited by commercial fisheries due to the associated richness and abundance of fish of high commercial value (Pitcher et al. 2007; Bergstad et al. 2012; Pham et al. 2015; Clark et al. 2016; D’Onghia 2019). New impacts derived from other increasing anthropogenic activities (Würtz and Rovere 2015; Rogers 2018) and the waste they generate (Pham et al. 2013; Woodall et al. 2015; Pierdomenico et al. 2019; Angiolillo and Fortibuoni 2020; Bo et al. 2021) also pose a challenge for the conservation of these ecosystems. More than 100 seamounts (and seafloor elevations) have been identified in the Mediterranean Sea (Morato et al. 2013; Würtz and Rovere 2015), which is one of the most threatened basins in terms of biodiversity loss and human impacts (Coll et al. 2010; Katsanevakis et al. 2014; Deudero and Alomar 2015; Moullec et al. 2019). All studies exploring Mediterranean seamounts and seafloor elevations agree on their key role for the biodiversity and functioning of the deep-sea benthic and demersal communities at local and regional scales (Danovaro et al. 2010; Morato et al. 2013; de la Torriente et al. 2018; Wienberg 2019; Bo et al. 2021; Massutí et al. 2022; Puerta et al. 2022).

Indeed, the conservation of vulnerable marine ecosystems (VMEs) in the deep sea has become an important concern for multiple international organizations (UNGA 2006; FAO 2009; ICES 2017; GFCM 2009), urging governments and Regional Fishery Management Organizations (RFMOs) to take action to prevent further damage on these fragile ecosystems. In this sense, the General Fisheries Commission for the Mediterranean (GFCM) elaborated a list of VME indicator taxa and habitats, which included cold-water corals (CWCs; e.g., *Desmophyllum pertusum* Linnaeus, 1758, (synonym *Lophelia pertusa*), and *Madrepora oculata* Linnaeus, 1758) and octocorals (e.g., *Funiculina quadrangularis* Pallas, 1766), among others (GFCM 2017). Fishing activities frequently cause physical damage on these species, reducing the coverage/abundance of such habitat-structuring organisms and the associated demersal fish fauna (Bo et al. 2014; Clark et al. 2016; Pierdomenico et al. 2018; D’Onghia 2019 and references therein). Moreover, the accumulation of marine litter has been reported as an important source of impact for benthic organisms (Fabri et al. 2014; Vieira et al. 2015; Dominguez-Carrió et al. 2020; Grinyó et al. 2020). Thus, the characterization and mapping of deep-sea communities, and particularly of VME indicator taxa and habitats, in seamounts and seafloor elevations are of prime interest to devise appropriate measures for the management of marine biodiversity, for instance, through the establishment of closed areas for fishing (e.g., no-take zones, Fisheries Restricted Areas; FAO 2023; GFCM 2019).

Among the seafloor elevations known to date in the Alboran Sea (western Mediterranean Sea) (Würtz and Rovere 2015; Wienberg 2019), the Seco de los Olivos Bank (SdO; also known as Chella Bank) is considered a biodiversity hotspot (Lo Iacono et al. 2008, 2012; Pardo et al. 2011; de la Torriente et al. 2014, 2018). In 2014, it was established as a Site of Community Importance (SCI code ESZZ16003) of the Natura 2000 network of protected areas. This SCI harbors unique assemblages of habitat-forming benthic invertebrates, such as CWC reefs, coral gardens, or sponge aggregations (Lo Iacono et al. 2008, 2012; de la Torriente et al. 2014, 2018). Furthermore, it presents a relevant economic target for fisheries since it hosts commercial species such as the European hake *Merluccius merluccius*, the common octopus *Octopus vulgaris*, and the Norway lobster *Nephrops norvegicus*, being traditionally and currently subjected to fishing activities (Pardo et al. 2011; de la Torriente et al. 2014).

Despite the efforts of the aforementioned studies to characterize the diversity of deep-sea habitats and assemblages in SdO, there is still a paucity of quantitative information standardized to area coverage, as generally occurs for the deep sea (e.g., Corbera et al. 2019; Grinyó et al. 2020). Therefore, the aims of the present study are (1) to qualitatively and quantitatively characterize the composition and diversity of the deep-sea megabenthic and demersal fish assemblages in the less studied areas of the SdO (i.e., the western and eastern ridges) and (2) document the indicators of anthropogenic activity in these areas. Integrated studies considering different assemblages and components of the ecosystem, as well as the identification of VME indicators and actual human impacts at local scale, are key to understand ecosystem health and establish efficient measures for their management and conservation.

## Material and methods

### Study area

**Fig. 1:**
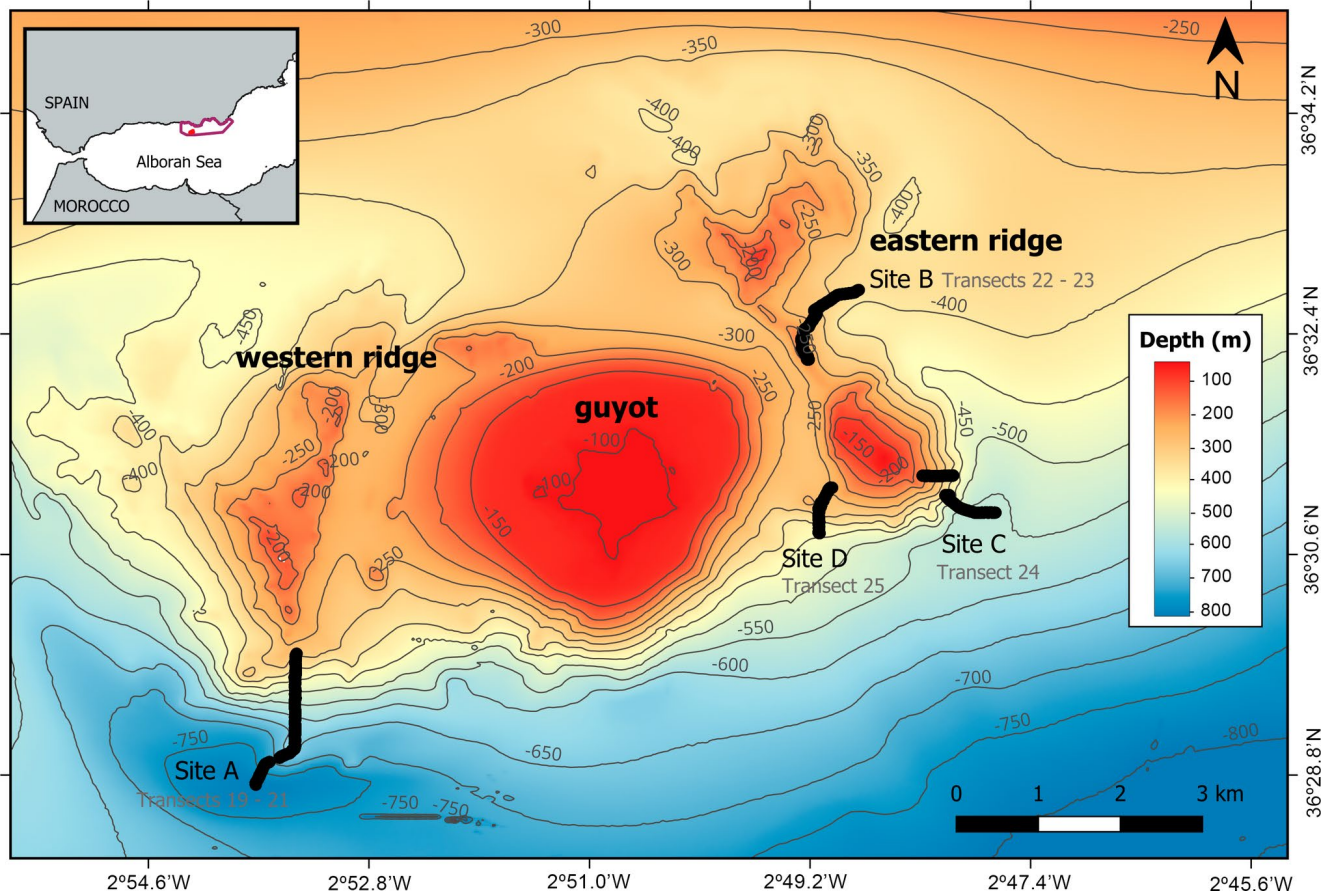
Location (inset map) and bathymetric map of the Seco de los Olivos Bank in the Alboran Sea, western Mediterranean. Depth isolines are shown in dark grey, while thick black lines represent the ROV transects performed during the MEDWAVES expedition. The purple polygon in the inset map indicates the limits of the Site of Community Importance of the Natura 2000 network *Sur de Almería—Seco de los Olivos*, where the study area is located (red dot)

The Seco de los Olivos Bank (SdO) is a seafloor elevation of volcanic origin located in the north-eastern sector of the Alboran Sea, in the western Mediterranean (Fig. 1) (Lo Iacono et al. 2012). This bank is represented by long ridges and several seafloor elevations, spanning an area of approximately 100 km^2^ (Maldonado and Comas 1992; Coiras et al. 2011), characterized by a high topographical complexity, as well as by a wide range of substrates and habitats (Abad et al. 2007; de la Torriente et al. 2014). The bank consists of three geomorphological structures (Fig. 1). The main structure is a “guyot” that rises approximately from 400 to 600 m depth, with a flat, semi-circular summit at ~ 75 m depth (De Mol et al. 2012; Lo Iacono et al. 2012; de la Torriente et al. 2019). The other structures are two ridges located on both sides of the main seafloor elevation (Fig. 1). The western ridge covers a depth range of 160–420 m and exhibits a N-S orientation in its southernmost area (Lo Iacono et al. 2012). It is composed of peaks and crests, and its flanks display an average slope of 8° with a maximum value of 17° (Lo Iacono et al. 2012). At the base of the ridge, a mix of mud and fine sands with some areas of coarse sands has previously been documented (Lo Iacono et al. 2012). The eastern ridge covers a depth range of 140 to 480 m along a NW–SE orientation (Lo Iacono et al. 2012), where dense frameworks of dead CWCs have been observed (Lo Iacono et al. 2012; de la Torriente et al. 2014).

The Alboran Sea is a transitional area where the Atlantic surficial inflow water and the Mediterranean deep-water mass flowing towards the Atlantic Ocean interact (Gascard and Richez 1985). The Modified Atlantic Water (MAW; 0–200 m depth) (Gascard and Richez 1985; Font et al. 1998; Millot 1999) mostly interacts with the summit of the guyot. At deeper layers, the Levantine Intermediate Water (LIW; 220–600 m depth) flows eastward throughout the Strait of Gibraltar, while the Western Mediterranean Deep Water (WMDW; > 600 m) (Gascard and Richez 1985; Parrilla et al. 1986) flows in the opposite direction.

### Data collection

The present study investigated the less studied areas of the SdO, which were sampled during the multidisciplinary expedition “MEDiterranean outflow WAter and Vulnerable EcosystemS” (MEDWAVES) on board of the R/V Sarmiento de Gamboa in 2016 (Orejas et al. 2017). This expedition was part of the H2020 European project “A Trans-Atlantic assessment and Deep-Water Ecosystem-Based Spatial Management Plan for Europe” (ATLAS, www.eu-atlas.org). The characterization of the deep-sea fauna was based on six video transects (Fig. 1; Table 1) conducted with the Remotely Operated Vehicle (ROV) Liropus 2000. It was equipped with a Kongsberg HDTW color zoom video camera (OE14-502), a Kongsberg underwater photo still camera (OE14-408/408E), and three frontal flash LED Matrix lights. Furthermore, a high definition (HD) video camera was used to obtain high-quality video footage. A pair of parallel laser beams, separated by 10 cm, were mounted on the ROV to provide scale to determine the area of the seafloor surveyed and for measuring organisms. The positioning of the ROV was recorded continuously using a USBL transponder and the HYPACK software. Further technical details can be found in Orejas et al. (2017), Mosquera-Giménez et al. (2019), and Puerta et al. (2022). A total of 18 h of video footage were recorded, which covered approximately 7 linear km of seafloor (Table 1).

**Table 1:** Specifications of the ROV video transects performed in the Seco de los Olivos Bank during the MEDWAVES expedition, including surveyed site, ROV transect according to the codes used in the MEDWAVES expedition (Orejas et al. 2017), date, geographical location (start and end latitude and longitude), duration of the video footage (hours, minutes, seconds), linear distance navigated in the transect (m), depth range (m), and orientation regarding the study area (flank). Sites include A, southern flank of the western ridge; B, middle portion of the eastern ridge; C, southeastern; and D, southwestern flanks of eastern ridge (as in Fig. 1)

Site	ROV transect	Date	Location (lat; lon)		Duration (hh:mm:ss)	Distance (m)	Depth range (m)	Flank
			Start	End				
A	19	23/10/2016	36° 7.98′ N; 2° 14.91′ W	36° 8.03′ N; 2° 14.88′ W	00:55:47	468	703–793	SE
	21	23/10/2016	36° 8.03′ N; 2° 14.87′ W	36° 8.28′ N; 2° 14.83W	04:05:08	1948	325–693	N
B	22	24/10/2016	36° 9.10′ N; 2° 13.55′ W	36° 9′ N; 2° 13.68′ W	03:10:05	1299	250–384	W
	23	24/10/2016	36° 9′ N; 2° 13.68′ W	36° 8.95′ N; 2° 13.67′ W	02:53:51	607	238–251	N
C	24	25/10/2016	36° 8.60′ N; 2° 13.23′ W	36° 8.68′ N; 2° 13.42′ W	03:32:18	1583	209–560	SE-W
D	25	25/10/2016	36° 8.55′ N; 2° 13.65′ W	36° 8.65′ N; 2° 13.62′ W	03:21:51	1125	282–446	N

According to the location of the video transects (Fig. 1), they were grouped into four different sites for their description and analysis. Site A includes transects 19 and 21 and is located on the southern flank of the western ridge. In contrast, Sites B, C, and D are located at the eastern ridge. Site B, in the middle portion, encompassing transects 22 and 23, while sites C and D in the southeast and southwest flanks of this ridge, respectively, account for only one transect each, numbers 24 and 25 (Fig. 1, Table 1).

### ROV video analysis

**Fig. 2:**
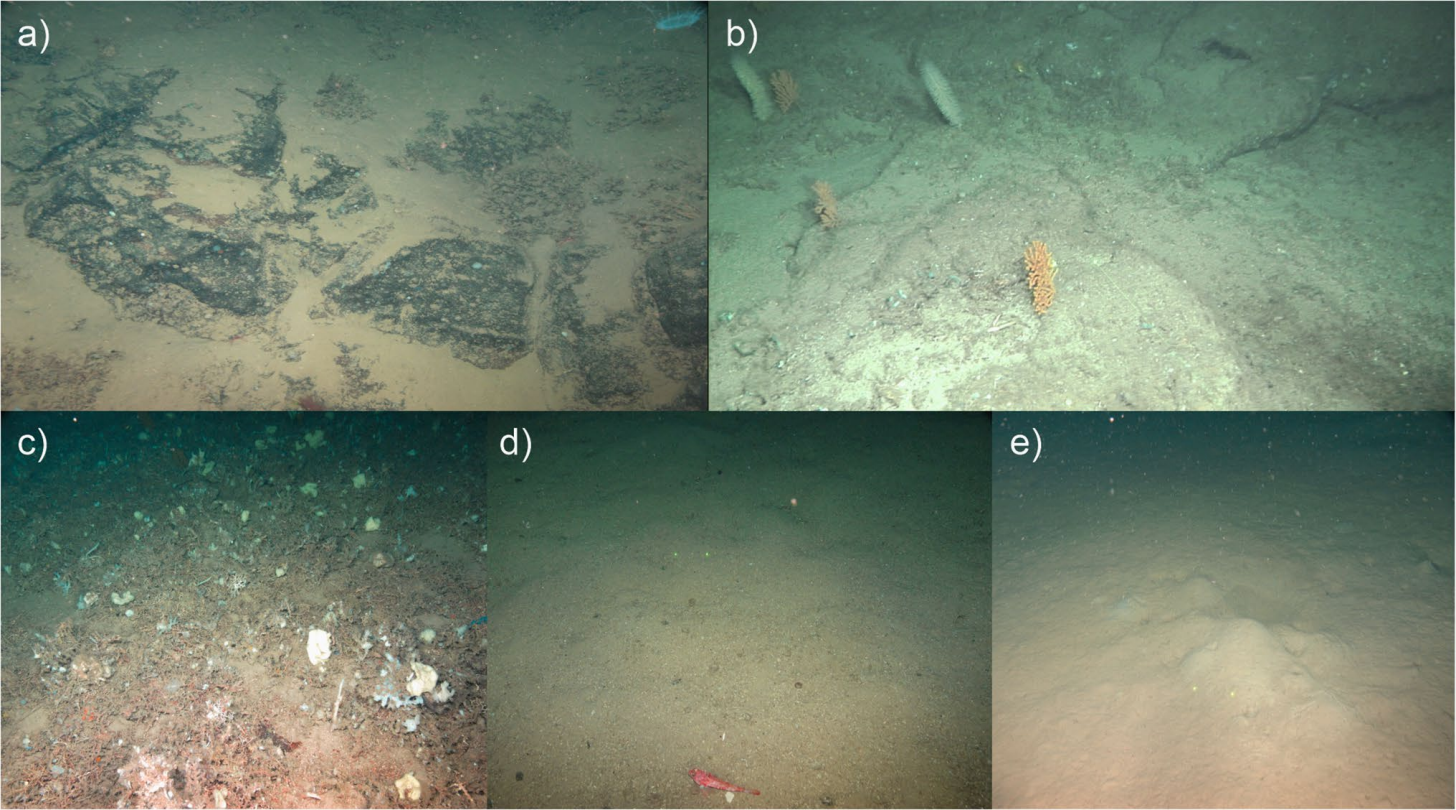
Reference images of the different substrate types identified at the Seco de los Olivos Bank. **a** Rock: bedrocks or large boulder outcropping. The rock surface is commonly covered by fine sediment; **b** flagstone: compact and consolidated sediment forming platforms or slabs that define a hard substrate, cracks are frequently present; **c** coral framework: substrate formed from the three-dimensional dead coral framework and/or accumulation of large broken pieces (> 5 cm) of this framework; **d** detritic unconsolidated sediment with large (> 0.5 cm approximately) grain size and frequently with bioclastic components (e.g., shells, coral rubble); **e** mud: unconsolidated sediment with visually undistinguishable grains, sometimes bioturbated

Prior to the video analysis, the complete footage from all transects was visualized to identify and classify the observed substrate types (Fig. 2), fauna (Suppl. Table S1, (Cañedo-Apolaya et al. 2020), and indicators of anthropogenic activities (e.g., bottom trawling marks). Substrate type characterization was based on a similar methodology as used in previous studies (Purser et al. 2013; van den Beld et al. 2017; de la Torriente et al. 2018; Puerta et al. 2022) and defined by five main categories: rock, flagstone (not volcanic origin), coral framework, which encompassed hard bottoms, and detritic and mud that formed the soft bottoms (Fig. 2).

The identification of deep-sea megabenthic and demersal fish fauna (Suppl. Table S1) (Cañedo-Apolaya et al. 2020) was solved to the lowest possible taxonomic level, using literature review (e.g., Xavier and Marzia 2017; Altuna and Poliseno 2019; Ordines et al. 2019; Rueda et al. 2019) and experts’ opinion. Some organisms could only be identified at high taxonomic levels (e.g., family, order) or as a descriptive morphotype (Cañedo-Apolaya et al. 2020). For these reasons, the Open Taxonomic Nomenclature described in Sigovini et al. (2016) and Horton et al. (2021) was used to name each taxon to the lowest possible taxonomical identification while indicating the level of uncertainty in such identification (Suppl. Table S1). No small encrusting organisms could be appropriately identified, and therefore, they were not included in the analyses.

The aim of the following analyses using ROV footage was to obtain standardized and comparable quantitative data across the sites surveyed to characterize the seafloor in terms of substrate type, megabenthic and demersal fish fauna, and anthropogenic impacts. For this purpose, video footage was processed following several steps: (1) selection of video material for standardized quantitative data and (2) annotations and quantification of substrate type, megabenthic fauna, and indicators of anthropogenic activity for the video material selected in the first step.

#### Selection of video material

For obtaining standardized quantitative data from the video material, the footage had to fulfill three criteria: (i) the ROV described (or navigated along) a linear path, (ii) image quality was good enough to clearly identify megabenthic and demersal fauna, and (iii) laser beams were clearly visible. The footage that met such requirements was retained for further analyses, with the remaining footage (e.g., ROV sampling, bad visibility, deployment, erratic movement) being discarded. From a total of 18 h of video footage, only 49% was selected for the subsequent analyses.

#### Video annotations

The video annotations recorded the presence of the different substrate types, organisms, or indicators of anthropogenic activity in relation to the position of the ROV and depth. Annotations were recorded using laser beams to define an (imaginary) horizontal reference line of 1 m width (see Suppl. Fig. S1). This width avoids using blurred edges of the video frames and allows the standardization of annotation to a given sampling area. This method was tested for bias in annotations by comparing records inside the reference line and in the full video frame (i.e., inside plus outside the reference line), showing no differences in the abundance and relative taxa composition patterns (Suppl. Fig. S2). All video annotations were performed using the Ocean Floor Observation Protocol (OFOP) v3.3.6 software, which links the position of the ROV with every second of the video footage. The georeferenced annotations for substrate coverage and fauna abundance logged per second were finally standardized to sampling units of 10 m^2^ (10.09 ± 0.49 m^2^).

First, video annotations for substrate types were performed on the selected video material described above. A maximum of two substrates were annotated at a time along the reference line. When a clear dominance of one substrate type was observed, a 100% coverage was assigned. The presence of a secondary substrate type was only annotated when it represented > 30% and ≤ 50%. However, to simplify and avoid complex substrate compositions, the annotation always assumed a fixed percentage of coverage (70% primary and 30% secondary) in the case that two substrate types were present.

Secondly, all organisms larger than 5 cm were identified and annotated (e.g., Puerta et al. 2022). The only exception to this criterion was Suberitidae gen. indet. (Suppl. Table S1) (Cañedo-Apolaya et al. 2020), which despite being sometimes smaller than 5 cm, it was always clearly identifiable, and hence annotated. Fauna annotations along the 1-m reference line were used for density calculations in sampling units of 10 m^2^, as individuals or colonies per square meter (ind·m^−2^) for each taxon.

Finally, the presence of items or indicators of anthropogenic activities was recorded. The abundance of indicators of anthropogenic activities was calculated as items per linear distance (km^−1^) as described in Angiolillo and Canese (2018) and grouped into four categories following the methods in Vieira et al. (2015): marine litter (e.g., cans, pieces of plastic), fishing remains (e.g., fishing lines, ropes, buoys, nets and traps), trawl marks, and other unidentified items.

### Data analyses

#### Composition and structure of deep-sea megabenthic and demersal fish assemblages

The composition and structure of the deep-sea assemblages at the SdO were explored through a cluster analysis to identify groups of associated fauna, including megabenthic (sessile or low mobility) and demersal fish (mobile) fauna. Square root transformed density data and a Bray–Curtis similarity matrix were used to build a hierarchical dendrogram applying the pair-group method using arithmetic averages (UPGMA), using site and substrate type as factors defining the groupings (Clarke et al. 2008). A similarity of percentage (SIMPER) analysis was performed to identify the taxa that most contributed to the intra-group similarity and the inter-group dissimilarity (i.e., differences among assemblages) (Clarke 1993). Taxa annotated less than five times along whole video footage were removed from the analyses, as well as other five taxa suspected of possible underestimation due to their size, characteristics, or behavior (i.e., *Munida* spp. indet., *Plesionika* spp. indet., *Paguridae* spp. stet., Suberitidae gen. indet., and *Hymenocephalus italicus*). Therefore, a total of 41 taxa were considered for these statistical analyses, which were carried out using the software PRIMER v.6 (Clarke and Gorley 2006).

#### Spatial diversity

Diversity was quantified and compared across the four sites explored using Hill numbers (Chao et al. 2014a) and their corresponding rarefaction and extrapolation (R/E) curves (Chao et al. 2014b). Hill numbers are a mathematical family of diversity indices, which are expressed as effective numbers of species or taxa (i.e., the number of species that constitute a hypothetical perfectly even community that presents the same diversity than the studied community). All of them were calculated under the same mathematical function (Chao et al. 2014a) but differed in the order *q*, which is sensitive to the frequency of occurrence of the species (Budka et al. 2019). The three most common *q* values were used, which are equivalent to three widely used diversity indices: species richness (*q* = 0), Shannon–Wiener diversity (*q* = 1, i.e., the effective number of abundant species), and inverse Simpson diversity (*q* = 2, i.e., the effective number of highly abundant species). In addition, rarefaction and extrapolation methods allowed to standardize the diversity measures and compare datasets with uneven sample sizes (Chao et al. 2014b). Following the approach in (Colwell et al. 2012), the R/E curves and their 95% confidence intervals were extrapolated to double the sample size of each site respectively. All (61) taxa were included in these analyses, which were performed using the R package iNEXT (Hsieh et al. 2016).

## Results

**Fig. 3:**
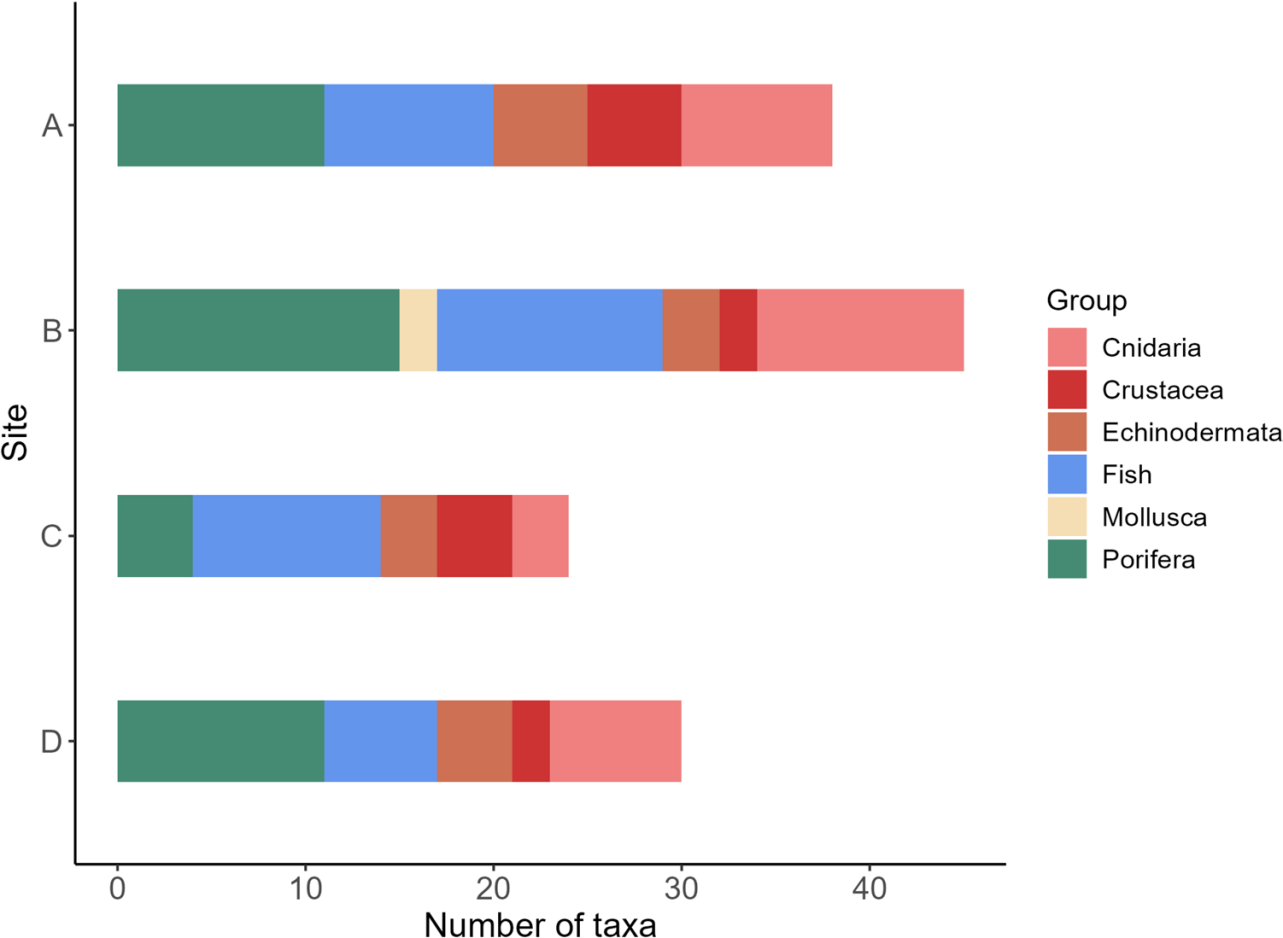
Taxonomic composition of the megabenthic and demersal fish fauna at each of the surveyed sites in the study area. Sites include **A**, southern flank of the western ridge; **B**, middle portion of the eastern ridge; **C** southeastern; and **D**, southwestern flanks of eastern ridge (as in Fig. 1)

A total of 1744 organisms were annotated from the selected video material, belonging to 61 taxa (Suppl. Table S1). Porifera (25%), Cnidaria (20%), and fish (29%) were the dominant groups with similar overall contribution to the taxonomic composition (Fig. 3). Comparing the different sites explored, site A showed the highest number of taxonomic groups, while site B presented the highest number of taxa and occurrences. In contrast, site C contained the lowest number of taxa.

The most frequent sponge taxa were Porifera fam. inc. sp.1 (255 organisms, 107 occurrences) and Porifera fam. inc. 1 (172 organisms, 114 occurrences). Among the diverse array of cnidarians, the gorgonian *Acanthogorgia* spp. indet. was the most abundant (135 colonies, 76 occurrences), followed by the black coral *Parantipathes larix* Esper, 1788 (82 colonies, 69 occurrences) and the stony coral *Dendrophyllia cornigera* Lamarck, 1816 (79 colonies, 58 occurrences). Regarding the ichthyofauna, the most frequent taxa were the blackbelly rosefish *Helicolenus dactylopterus* Delaroche, 1809 (94 individuals, 92 occurrences) and the slime-head fish *Hoplostethus mediterraneus* Cuvier, 1829 (72 individuals, 20 occurrences), which formed a large shoal at the western ridge.

**Fig. 4:**
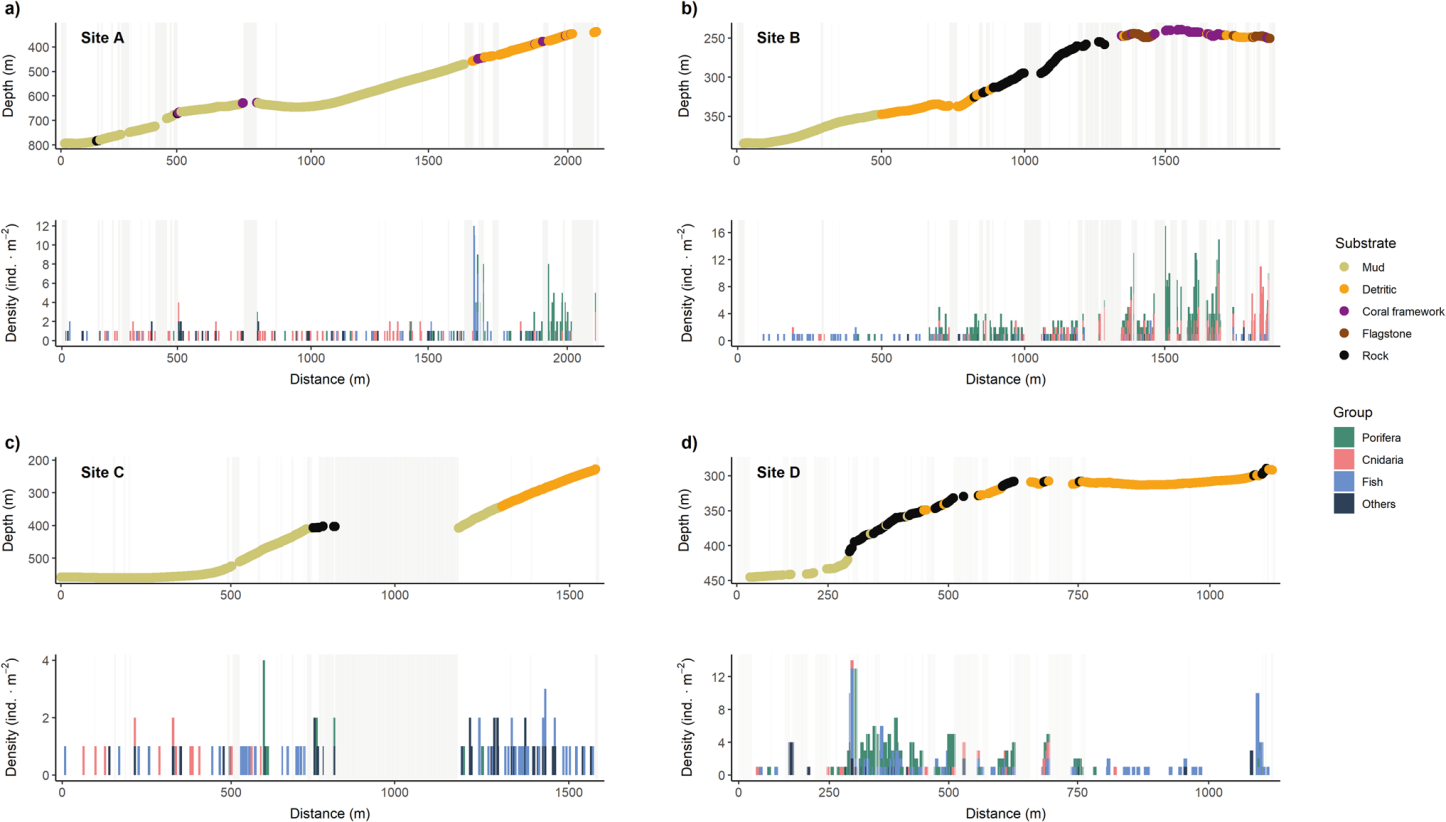
Characterization of substrate type and megabenthic and demersal fauna recorded along the ROV transects performed at the four studied sites in the Seco de los Olivos Bank. Panels **a**, **b**, **c**, **d**, represent the corresponding Sites A, B, C, D as in Fig. 1. At each panel, the upper plot shows substrate type along the transect depth profile, while the lower plot shows the density of organisms grouped by the main taxonomic groups along the distance navigated in the transect (m). Shadow grey areas denote parts of the transects that were not analyzed as did not meet selection criteria for quantitative analyses

Almost 98% of the footage analyzed was characterized by a unique dominant substrate type. Overall, soft substrates were widely distributed at all sites and depths, accounting for 84% of the bottom composition (Fig. 4). Hard substrates occurred in patches at sites B and D above 400 m depth. Coral framework was occasionally detected in the shallowest areas of sites A and B in (239–251 m depth) (Fig. 4).

### Site A: southern flank of the western ridge

Site A was surveyed by ROV transects 19 and 21 (Table 1), covering the deepest areas (338–722 m depth), and located on the southern flank of the western ridge (Fig. 1). The seafloor was primarily characterized by mud, except in some sections of the shallowest part of the transect (~ 340–450 m depth), where detritic substrates occurred with some patches of coral framework (Fig. 4a). The megafauna was dominated by Porifera, Chordata (fish), and Cnidaria in terms of number of taxa. Sponges were distributed mostly along the shallowest part of the transect (350–500 m depth), with Porifera fam. inc. sp.1 being the most common taxa, mainly found on coral framework (0.32 ± 0.79 ind·m^−2^) and detritic (0.10 ± 0.57 ind·m^−2^) substrates. Cnidaria was the most common group below 500 m depth (Fig. 4a), with high densities reported for the seapens *Kophobelemnon stelliferum* and *Pennatula* cf. *aculeata*. Other invertebrates observed in the deepest section on soft substrates included decapod crustaceans (e.g., squat lobsters *Munida* spp. indet., pandalid shrimps *Plesionika* spp. indet, Suppl. Fig. S2) and echinoderms (Fig. 4a). Single observations of the echiurid (Polychaeta) *Bonellia* cf. *viridis* and the bivalve (Mollusca) *Neopycnodonte zibrowii* Gofas, C. Salas & Taviani, 2009, were registered. Ray-finned fishes were observed along the entire transect (Fig. 4a), while a large shoal of the slime-head fish *H. mediterraneus* was observed over small patches of coral framework. Other fish species such as the blackbelly rosefish *H. dactylopterus* and the macrurid *Nezumia aequalis* Günter, 1878, were commonly detected as solitary individuals along with sporadic sightings of other fishes (e.g., *Capros aper* Linnaeus, 1758, *Hymenocephalus italicus* Giglioli, 1884, *Phycis blennoides* Brünnich, 1768, and sharks of the genus *Galeus*).

### Site B: middle portion of the eastern ridge

The ROV transects 22 and 23 (Table 1) were conducted at site B. This site is located in the middle portion of eastern ridge, which connects two small banks (Fig. 1). Transect 23 covered the shallowest sections (239–251 m depth) where hard substrates (i.e., coral framework, flagstone and rock) dominated (Fig. 4b). Downslope, transect 22 surveyed the deepest section (255–384 m depth), and was characterized by rocks that turned into soft bottoms with depth (Fig. 4b). This site presented the highest densities of habitat-forming taxa, such as sponges and corals, which usually co-occurred mainly associated with hard substrates (Fig. 4b). The sponges Porifera fam. inc. sp.1 (1.22 ± 2.27 ind·m^−2^ on coral framework) and Porifera fam. inc. 1 (0.12 ± 0.55 ind·m^−2^ on coral framework) were the most abundant among the 14 Porifera taxa observed. Other common sponges were *Cladocroce* spp. indet. (which probably includes *Cladocroce fibrosa* Topsent, 1890), Porifera fam. inc. sp.4 and *Asconema setubalense* Kent, 1870. The gorgonian *Acanthogorgia* spp. indet. was commonly found on all substrates except mud, reaching the highest densities on flagstone (0.62 ± 1.48 ind·m^−2^). The black coral *P. larix* and the stony coral *D. cornigera* occurred across all substrate types, with maximum densities observed on rocks (0.13 ± 0.42 col m^−2^) and coral framework (0.32 ± 0.72 col·m^−2^), respectively. A few observations of small scleractinian colonies (i.e., *M. oculata D. pertusum*), gorgonians (e.g., *Savaglia savaglia* Bertoloni, 1819, *Callogorgia verticillata* Pallas, 1766), and sea pens (e.g., *F. quadrangularis)* were also recorded. Among other invertebrates, the gastropod *Ranella olearium* Linnaeus, 1758, and the octopus *Eledone cirrhosa* Lamarck, 1798, were only found in this site. Fish were observed in low abundances across the entire transect, being *H. dactylopterus* the most frequent, followed by *Gadiculus argenteus* Guichenot, 1850, *Anthias anthias* Linnaeus, 1758, *C. aper*, *Conger conger* Linnaeus, 1758, and the sharks *Galeus* sp. indet. and *Scyliorhinus canicula* Linnaeus, 1758 (Fig. 4b).

### Site C: southeastern flank of the astern ridge

At the southeast flank on the eastern ridge (228–560 m depth), ROV transect 24 (Table 1) was carried out to survey site C (Fig. 1). This site was mainly characterized by mud, with an area of detritic substrates in the shallowest section (~ 250–350 m depth) (Fig. 4c). This area presented the lowest densities of organisms (Fig. 4c), being Heteroscleromorpha fam. indet and ceriantharians (Cnidaria, Ceriantharia fam. indet) (Suppl. Fig. S2) the most common taxa. Other invertebrates such as pandalid shrimps or sea urchins (Suppl. Fig. S2) were occasionally observed. However, the number of fish taxa recorded was high (9 in total), being *H. dactylopterus*, *Coelorinchus caelorinchus* Risso, 1810, and *H. italicus*, the most frequent (Fig. 4c).

### Site D: southeastern flank of the eastern ridge

Site D was explored by ROV transect 25 (290–445 m depth; Table 1) at the southwest flank of the astern ridge (Fig. 1). The substrate composition largely varied across the transect, being dominated by detritic substrates at the shallowest section (Fig. 4d), rocks between 330 and 400 m depth, and mud in the deepest section (Fig. 4d). The highest densities of fauna were mainly observed on rocky substrates in a similar depth range. The three main groups of fauna (Porifera, Cnidaria, and fish) were commonly detected, with several Porifera taxa, including *A. setubalense* (0.13 ± 0.41 ind·m^−2^) frequently found on rocks (Fig. 4d). Scattered colonies of *Acanthogorgia* spp. indet. and *P. larix* were also observed on hard bottoms. A smaller number of other invertebrates were registered compared to other sites, being the sea urchin Cidaridae spp. indet. the most abundant as in the rest of sites. Fish such as *H. dactylopterus* were widely distributed across site D, whereas the blue whiting *Micromesistius poutassou* Risso, 1827, was detected only on soft substrates (Fig. 4d).

### Deep-sea megabenthic and demersal fish assemblages

**Fig. 5:**
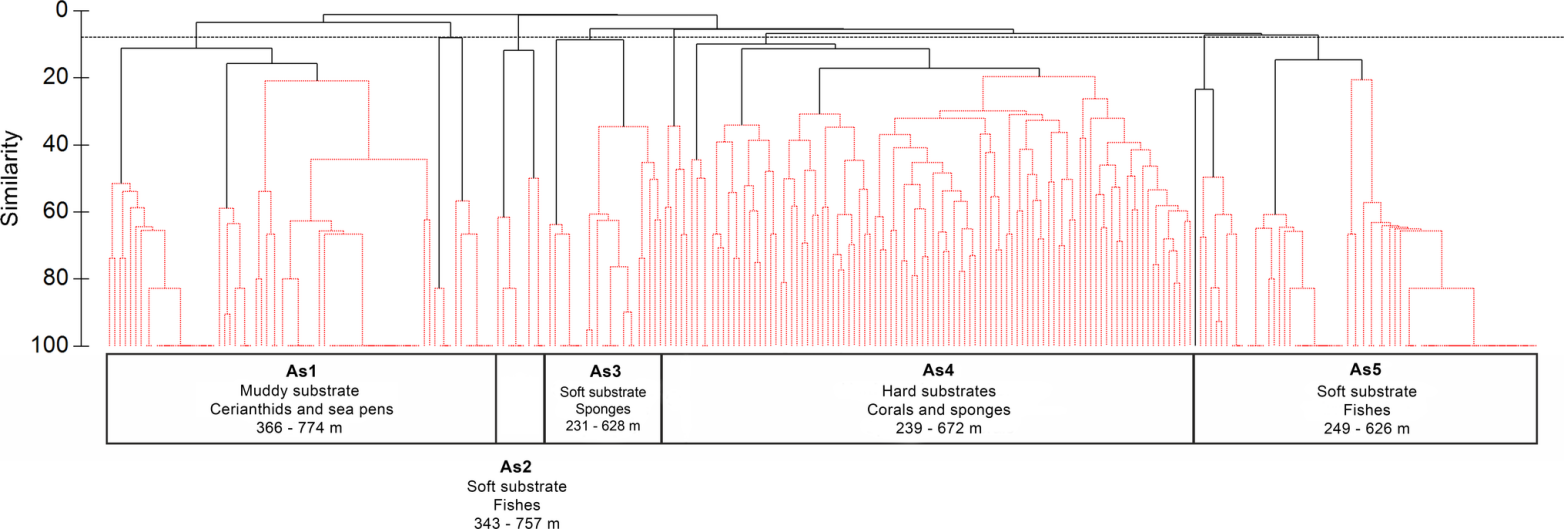
Hierarchical clustering for deep-sea megafauna of the Seco de los Olivos Bank. Black dashed line indicates the reference similarity percentage used to delimit the major assemblages. Each assemblage is labeled with As and number (e.g., Assemblage 1 = As1), depth range, dominant substrate, and the megafauna explaining at least 60% of the intragroup similarities

**Fig. 6:**
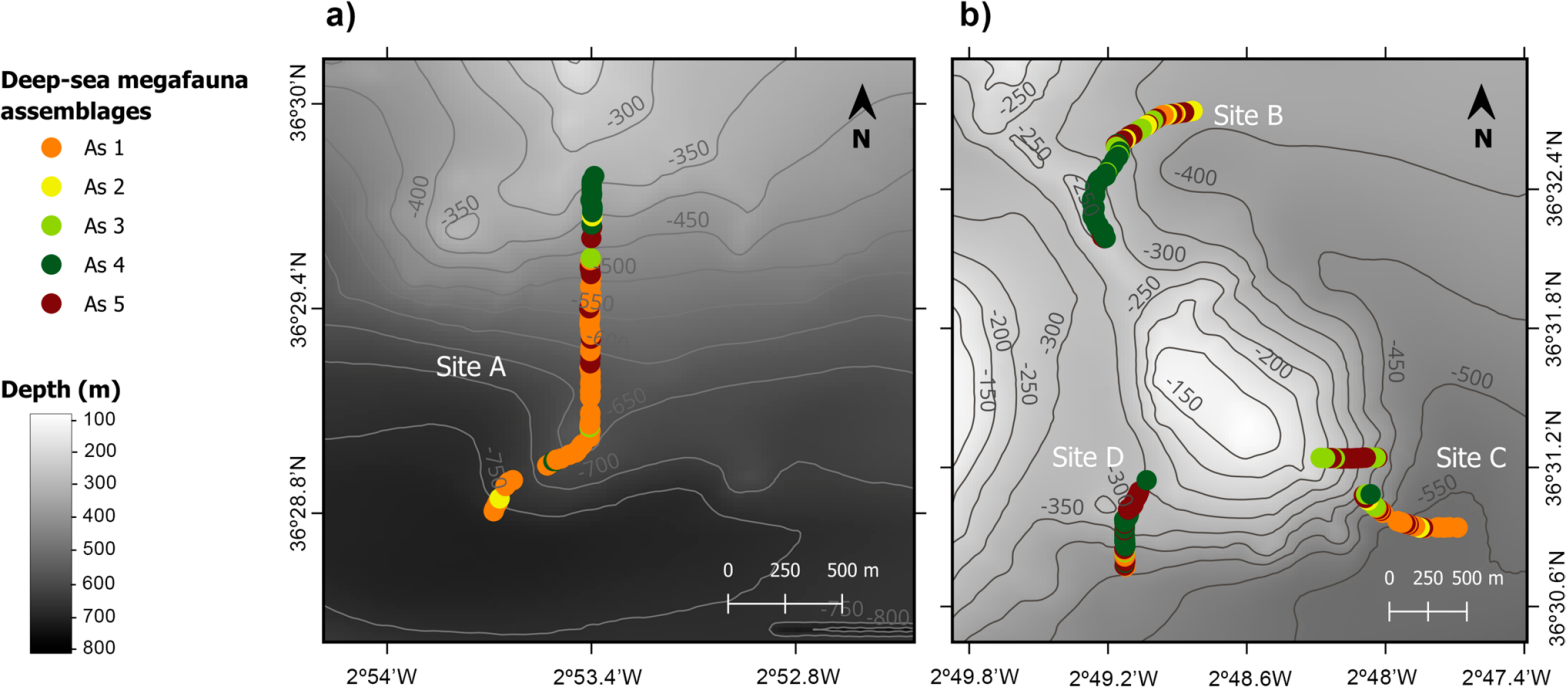
Spatial distribution of the five deep-sea megafauna assemblages identified in the study area. **a** Site A, southern slope of the western ridge. **b** Included sites B, middle portion of the eastern ridge, C southeastern, and D, southwestern flanks of eastern ridge (as in Fig. 1). See Fig. S3 for the characterization of the assemblages

The hierarchical cluster analysis identified five major benthic and demersal fish assemblages, mainly defined by species composition and related to substrate type (Fig. 5). The factor site displayed a minor effect in the groupings, and it was only detected through the scaling up of the dendrogram. Overall, sponges, cnidarians, and fishes were the most abundant groups in the analyses and, thus, the most representative taxa typifying each assemblage (Suppl. Table S2, Fig. S3). The geographical location of each assemblage along the video transects is shown in Fig. 6. In addition, the SIMPER analysis highlighted differences between these assemblages with high levels of dissimilarities, ranging from 93.2 to 99.8% (Suppl. Table S2). Within each assemblage, the average similarity showed low values (21.1–43.8%; Suppl. Table S2), and usually, one or two taxa explained more than 60% of the intra-group similarity (Suppl. Table S2).

Assemblage 1 (As1: *Muddy bottoms with cerianthids and sea pens*) inhabited mud plains between 366 and 774 m depth, in sites A and C (Figs. 5 and 6, Suppl. Fig. S3). Two cnidaria, Ceriantharia fam. Indet. and *K. stelliferum*, which showed similar densities, explained almost 90% of the intra-group similarity (Suppl. Table S2). Assemblage 2 (As2: *Soft bottoms dominated by G. argenteus*) was mainly observed on soft substrates at sites A, B, and C, covering a bathymetric range of 343–757 m depth (Figs. 5 and 6, Suppl. Fig. S3). The total intra-group similarity of As2 (Suppl. Table S2) was explained by the presence of two ray-finned fishes, the silvery pout *G. argenteus*, and the blackspot seabream *Pagellus bogaraveo* Brünnich, 1768*.* Assemblage 3 (As3: *Mixed assemblage of small sponges and Callionymidae on soft bottoms*) occurred mainly on mud and detritic substrates between 231 and 629 m depth, but it was also observed sporadically on patches of rocks and coral framework. This assemblage was commonly found at sites B and C (Figs. 5 and 6, Suppl. Fig. S3), being the only dominated by both invertebrates and fish (Suppl. Table S2), including the sponge Heteroscleromorpha fam. indet. and fish from the family Callionymidae (Callionymidae spp. indet). Assemblage 4 (As4: *Hard bottoms with habitat-forming Porifera and Cnidaria*) mainly occurred on hard substrates at a depth of 239–672 m (Figs. 5 and 6, Suppl. Fig. S3). Multiple taxa typified this assemblage, with a majority of habitat-forming (Suppl. Table S2) such as Porifera, which explained 42.52% of the intra-group similarity, as well as different types of corals (e.g., *P. larix, D. cornigera*) that explained 19.36%. Massive sponges (Porifera fam. inc. 1, Porifera fam. inc. sp.1) formed dense aggregations only at site B and over coral framework. Finally, assemblage 5 (As5: *Soft bottoms dominated by H. dactylopterus*) was associated with soft substrates across all sites and distributed from 259 to 626 m depth (Figs. 5 and 6, Suppl. Fig. S3). This assemblage was primarily characterized by two ray-finned fishes, *H. dactylopterus* and *C. caelorinchus*, explaining almost all the intra-group similarities (Suppl. Table S2).

### Spatial diversity

**Fig. 7:**
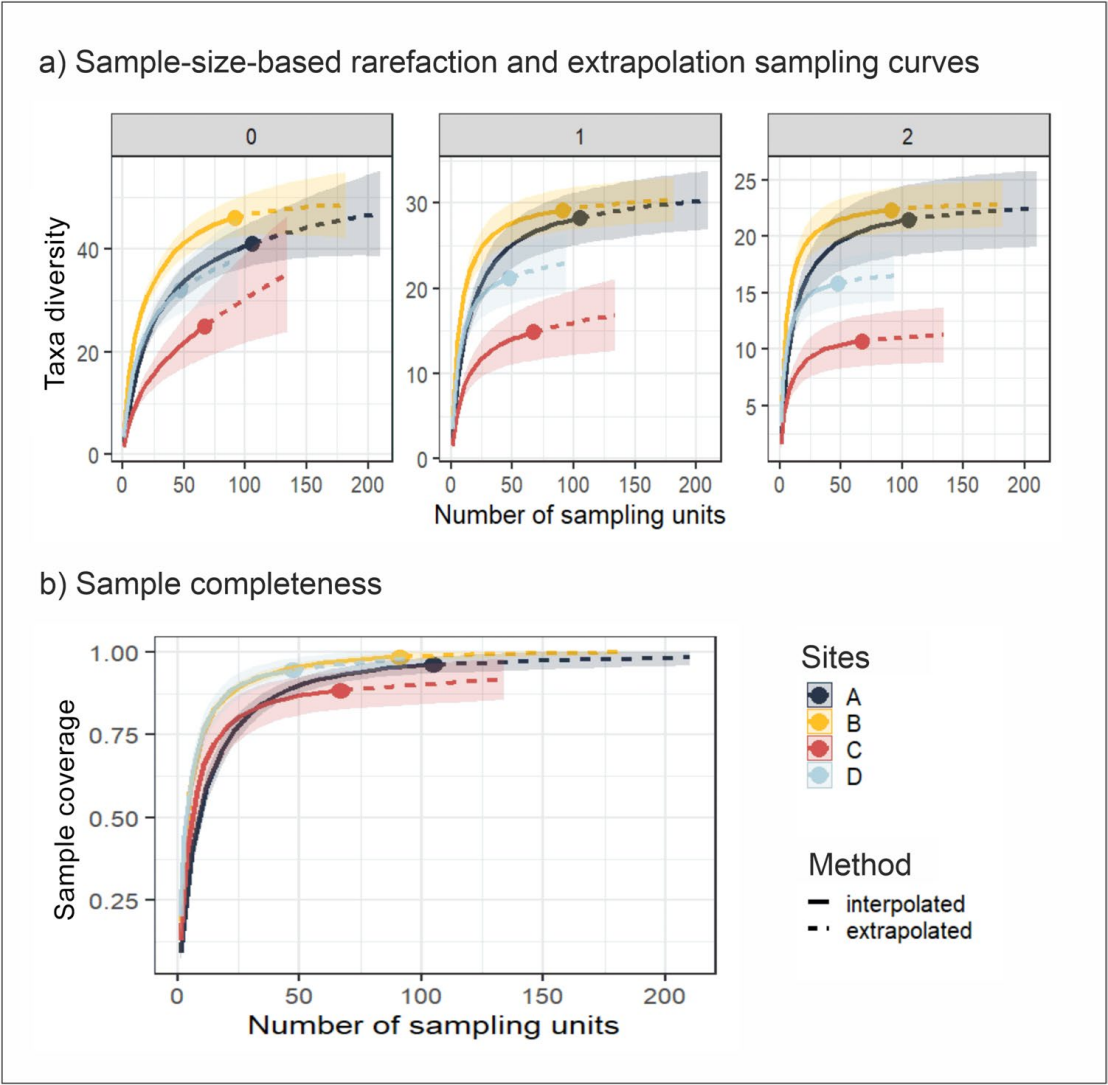
Rarefaction (solid line)/extrapolation (dashed line) curves of diversity at the studied sites in the study area based on Hill numbers. **a** R/E curves based on most common Hill numbers calculated for order *q* = 0, 1, and 2; **b** total sample coverage curve against number of sampling units

Comparisons of diversity indices based on R/E curves of Hill numbers at same number of sampling units indicates that site B was the most diverse in terms of species richness (*q* = 0), followed very closely by site A, which could even surpass it if extrapolated to larger number of sampling units (Fig. 7). By contrast, site C displayed the lowest diversity in all the indices (Fig. 7). It should be noticed that site B presented the highest (98.4%) sample coverage (i.e., more sample units than other sites but site A) and site C the lowest (90.1%) (Fig. 7). In addition, the asymptotic R/E curves did not completely stabilize at a fixed value of the corresponding diversity in all cases, which might indicate a low number of samples to calculate true diversity indices. Also, the R/E curves presented wide confidence intervals, which overlap among some of the sites, indicating non-significant differences in average diversity values among these sites. However, significant differences in the effective number of taxa became clearer beyond ~ 50 sampling units and mainly between the most (sites A and B) and the least (sites C and D) diverse sites (Fig. 7).

### Quantification and distribution of indicators of anthropogenic activities

Signs of anthropogenic activities were observed at the four sites explored in the SdO and across the complete bathymetrical range studied (Suppl. Fig. S4). Overall, remains of fishing gears (62 items in total) were the most commonly observed category (69% of the total; Table 2), mainly including remains of different fishing lines (Suppl. Fig. S4) and occasionally ropes, buoys, and a trap. The size of the fishing lines remains ranged from 50 cm to more than 1 m long. Bottom trawling marks (11 occurrences in total) were found on mixed and soft bottoms (sites B and C) as parallel lines marked on the substrate of approximately 15 cm wide each. Marine litter (14 items in total) mainly encompassed glass bottles followed by plastic objects and metal cans (Suppl. Fig. S4; Table 2). Very few signs of anthropogenic activities were observed at site A, while most occurred at sites D and B (29 and 39 items in total, respectively). Signs of anthropogenic activities were unevenly distributed among the megafauna assemblages previously identified (Table 2).

**Table 2:** Quantification of indicators of anthropogenic activities (items·km^−1^) for each of the assemblages (see Fig. 5 for details) in the Seco de los Olivos Bank and the relative contribution (percentage, %) to the total observations

Anthropogenic impact		As1	As2	As3	As4	As5	Overall	Percent
Fishing remains	Buoy	1.35			1.04		22.34	69.32
	Line	1.35	20	27.27	39.58	13.64		
	Rope				2.08			
	Trap					1.52		
Marine litter	Glass			4.55	3.13	4.55	4.76	14.77
	Metal		10	4.55	1.04	1.52		
	Plastic	1.35			1.04			
Others		1.35		4.55	4.17		2.2	6.82
Trawl marks			20	9.09		6.06	2.93	9.09
**Total**		5.41	50	50	52.08	27.27		
**%**		2.93	27.06	27.06	28.19	2.93		

Thus, assemblages As2 and particularly As4 accounted for most of the indicators of anthropogenic activities (Table 2). A similar number of items were observed in the most impacted assemblages (as2, As3, As4), with the majority corresponding to remains of fishing gears. The presence of anthropogenic activities was highest in As4, with a large number of fishing lines (Table 2). Bottom trawling marks occurred in all assemblages dominated by fish species (As2, As3, As5).

## Discussion

Despite the ecological importance and recognition of the SdO (also known as Chella Bank), the characterization and the functioning of this key area for marine biodiversity of the western Mediterranean has not been completely understood to date. The present work complements and updates the characterization of the deep-sea megafauna composition, structure, and diversity across some of the least explored areas within the SdO (de la Torriente et al. 2014, 2018, 2019, 2022), providing new quantitative data on some key deep-sea taxa. Still, less than 15% of Mediterranean seamounts have been biologically sampled or at least visited once (Morato et al. 2013; Würtz and Rovere 2015), and few studies reported quantitative data on deep-sea benthic communities (Orejas et al. 2009; Cau et al. 2015; Corbera et al. 2019; Enrichetti et al. 2019; Grinyó et al. 2020; Dominguez-Carrió et al. 2022) limiting the understanding and assessment of these ecosystems.

The 62 taxa identified in this study (Suppl. Table S1) were reported in previous works conducted in the study area (de la Torriente et al. 2014; Puerta et al. 2022) and other seafloor elevations in the surrounding areas of the Alboran Sea (Abad et al. 2007; Hebbeln et al. 2009; Pardo et al. 2011; Würtz and Rovere 2015; Corbera et al. 2019; Grinyó et al. 2020; Lo Iacono et al. 2020). Among these geomorphological features, SdO showed the highest taxa richness, even considering the differences in the surveyed area. Our results showed that taxa diversity, composition, and structure of deep-sea megafauna in SdO were strongly influenced by the patchy distribution of substrate types as previously reported (de la Torriente et al. 2018; Puerta et al. 2022) and also described in other seafloor elevations (Auster et al. 2011; Zeppilli et al. 2016; Ramalho et al. 2020; Urra et al. 2021). This high biodiversity has been related to the presence of dense colonies and coral framework of *M. oculata* and *L. pertusa* (Hebbeln et al. 2009; Pardo et al. 2011; De Mol et al. 2012; Corbera et al. 2019; Wienberg 2019; Ramalho et al. 2020) that colonize hard bottoms. This type of substrate was widely proven to be able to host higher biodiversity (e.g., Howell et al. 2010). In the present study sites, A and B were dominated by patches of different hard substrates (i.e., rock, flagstone, and coral framework), providing a more complex and heterogeneous three-dimensional structure of the physical habitat that can host higher diversity of species. Thus, these sites showed higher biodiversity than the southernmost location (site A) dominated by mud. However, the complex hydrodynamics, with strong currents, eddies, and upwelling process in the Alboran Sea (Vargas-Yáñez et al. 2020) that enhance food availability (Oguz et al. 2014; García-Martínez et al. 2019), are likely to be key to maintain these highly diverse ecosystems (Abad et al. 2007; De Mol et al. 2012; de la Torriente et al. 2019).

The five assemblages found and described here matched some of those previously described by (de la Torriente et al. 2014, 2018, 2019). However, the typifying taxa of each assemblage differ at low taxonomic levels. Such differences might be explained by the much larger sampling effort (55 ROV transects, 76–700 m depth) in previous studies (de la Torriente et al. 2014, 2018, 2019), which provided a comprehensive overview of the different habitats and the geomorphological structures at the bank and/or differences in the methodologies applied. In addition, both the previous and the present studies highlighted the difficulties in taxonomic identification using mainly imagery methods (Henry and Roberts 2014; Woodall et al. 2018; Howell et al. 2019). In this sense, the standardized Open Nomenclature (Sigovini et al. 2016; Horton et al. 2021) used in this study will facilitate comparability with existing and future studies and databases.

Despite potential methodological differences between studies, As1 detected in the present study clearly corresponds to “Habitat 10” described by de la Torriente et al. (2018, 2019), which was typified by the sea-pen *K. stelliferum* on mud. As4 characterized by high abundances of benthic suspension-feeding and habitat-forming organisms (mainly sponges, corals, and gorgonians) on hard substrates seems to be a counterpart of the “Habitat 8” (corals) and “Habitat 9” (gorgonians and sponges) detected in de la Torriente et al. (2018, 2019). Interestingly, the predicted distribution of “Habitats 8 and 9” (de la Torriente et al. 2019) is in agreement with the location of As4 in this study. This result further validates the outcomes of the predictive models in (de la Torriente et al. 2019) with independent data, highlighting the ability and utility of species distribution models for habitat mapping in unexplored or data-poor areas (Araújo et al. 2019), which is critical for the application of management and conservation initiatives (Davies and Guinotte 2011; Anderson et al. 2016; Standaert et al. 2023; Georges et al. 2024). Besides the main structuring species, no other typifying taxa of the assemblages identified here were representative in the habitats described by de la Torriente et al. (2018, 2019). However, the fishes *G. argenteus* and *H. dactylopterus* were also reported as very abundant among the ichthyofauna on coarse sandy areas of SdO along a wide bathymetric range (100–500 m and 100–700 m depth, respectively) (Abad et al. 2007; Pardo et al. 2011; de la Torriente et al. 2014). A clear and large variability was detected in the total length of *H. dactylopterus* (4.7–28.8 cm) across the observed organisms, which indicates the presence of small and large individuals (length at maturity ~ 14 cm) (Muñoz and Casadevall 2002). These observations highlight the role of SdO as a fish nursery area, as it was previously documented also for the species *M. merluccius* (Pardo et al. 2011; de la Torriente et al. 2014; Muñoz et al. 2018).

Nine taxa observed in this study (i.e., *D. pertusum*, *M. oculata*, *Acanthogorgia* spp. indet. *D. cornigera*, *P. larix*, *K. stelliferum*, *A. setubalense*, *Phakellia* sp., Malacalcyonacea spp. indet) were identified as VME indicator taxa (FAO 2009) and/or recognized as endangered species (i.e., *F. quadrangularis*, *D. diantus*) that require special protection by international agreements such as the EU Habitats Directive (92/43/CEE), the Barcelona Convention (UNEP/MAP-SPA/RAC 2018), the UICN (www.uicn.org, Suppl.  Table S1), or the Spanish law for conservation of the Natural Patrimony and Biodiversity (Law 42/2007). The defining characteristics of VME indicator taxa (FAO 2009), such as rarity, fragility, or importance for the ecosystem functioning, leave these organisms at significant risk from the impacts of human activities, including fisheries (FAO 2009), maritime traffic (de la Torriente et al. 2014; Coomber et al. 2016), or marine litter (Fabri et al. 2014; Angiolillo et al. 2015; Vieira et al. 2015; Pierdomenico et al. 2019).

For instance, Grinyó et al. (2020) reported a reduction in habitat complexity and local beta diversity caused by trawling in soft bottom areas of the Alboran Sea. As in other studies in the western Mediterranean (Fabri et al. 2014; Vieira et al. 2015; Pierdomenico et al. 2019; Dominguez-Carrió et al. 2020; Grinyó et al. 2020; Angiolillo et al. 2021), including SdO (De la Torriente et al. 2022), our results showed that anthropogenic impacts resulting from fishing activity were the most common, being present in all of sites and assemblages. The least diverse sites studied also accounted for the highest number of anthropogenic impacts.

Bottom trawling marks were mainly found in soft bottoms as this is the preferred substrate for this fishery. Indeed, soft bottom assemblages found here were mainly characterized by fish taxa. Therefore, the absence or low presence of sessile fauna in these bottoms suggests that bottom trawling could be removing and preventing the establishment of epibenthic megafauna. In contrast, habitat-forming assemblages showed the highest levels of disturbance due to the presence of fishing remains. Trawling and longlining have been strongly present in the SdO for several decades (de la Torriente et al. 2014, 2019, 2022; Samy-Kamal et al. 2014), with fishing fleets frequently operating in the SCI at least up to 2010 (De la Torriente et al. 2022). Trawling effort was mainly distributed north to the SdO and in the eastern ridge, while longlining was concentrated on the crests of both ridges (De la Torriente et al. 2022), coinciding with our observations of anthropogenic impacts. The damaging effects of fisheries and fishing remains are well known for benthic communities and particularly for habitat-forming species (e.g., D’Onghia et al. 2017; Pierdomenico et al. 2018; Angiolillo et al. 2021), which can even impact ecosystem functioning and productivity (Jennings et al. 2001) and also local fisheries (Victorero et al. 2018). The typical interactions between the anthropogenic remains and the megabenthic and demersal fauna (Pierdomenico et al. 2019; Angiolillo et al. 2021), such as large sponges over fishing lines, fish sheltering in litter or coral entangled by longlines (Suppl. Fig. S3), were observed also in this study. Nevertheless, it is very difficult to disentangle whether such interactions were recent or could indicate the recolonization of some organisms after an old impact event.

Under the SCI category, a preventive protection regime was established in SdO aiming to ensure no deterioration of the marine habitats. However, regulations of human activities are still pending. Efforts have been recently invested in the assessment of the “Good Environmental Status” of the Alboran Sea, including SdO under the Marine Strategy Framework Directive (2008/56/EC). However, these evaluations rarely apply to the deep-sea realm (Kazanidis et al. 2020; Orejas et al. 2020). Therefore, the present results contribute to assess and manage this key deep-sea ecosystem in the framework of the main European policies such as the Habitats Directive (92/43/CEE), the Marine Strategy Framework (2008/56/EC), and the Maritime Spatial Planning (2014/89/EU) Directives (Manea et al. 2020; De la Torriente et al. 2022), but also to advice international organisms such as the General Fisheries Commission of the Mediterranean to fulfill their aims of protecting sensitive habitats and species (GFCM 2009; 2019).

## Supplementary Information

Below is the link to the electronic supplementary material.

Supplementary file1 (DOC 3297 KB)

## Declarations

### Sampling and field studies

All necessary permits for sampling and observational field studies have been obtained by the authors from the competent authorities and are mentioned in the acknowledgements. The study is compliant with CBD and Nagoya protocols.

### Data availability

The video annotation datasets can be downloaded from PANGEA at (*currently in uploading and validation processes; link will be provided when available*).
