# Supplementary material for: Deep-sea ecosystems in the north-eastern Alboran Sea (western Mediterranean): quantifying assemblages and anthropogenic activity in the Seco de los Olivos Bank
Source: Mar Biodivers. 2025 Mar 22;55(2):31. doi: 10.1007/s12526-025-01505-4 (PMC11929697; doi:10.1007/s12526-025-01505-4)
Supplement: Supplementary file 1 — Supplementary file1 (DOC 3297 KB) [file 12526_2025_1505_MOESM1_ESM.doc]

**Supplementary Information**

**Deep-sea ecosystems in the north-eastern Alboran Sea (Western Mediterranean):** **quantifying assemblages and anthropogenic activity in the Seco de los Olivos Bank**

Patricia Puerta^a*^, Rosa M. Cañedo-Apolaya^ab^, José L. Rueda^c^, Carlos Dominguez-Carrió^de^, Javier Urra^c^, Covadonga Orejas^f^

^a^Centro Oceanográfico de Baleares, Instituto Español de Oceanografía - CSIC, Palma, Spain. ^*^Corresponding author: patricia.puerta@ieo.csic.es

^b^Department of Biology, Marine Biology, Ghent University, Gent, Belgium.

^c^Centro Oceanográfico de Málaga, Instituto Español de Oceanografía - CSIC, Málaga, Spain.

^d^Instituto de Investigação em Ciências do Mar – Okeanos, Universidade dos Açores, Horta, Portugal.

^e^IMAR Instituto do Mar, Universidade dos Açores, Horta, Portugal.

^f^Centro Oceanográfico de Gijón, Instituto Español de Oceanografía - CSIC, Gijón, Spain.

**Figure S1.** Graphical example of the video annotation approach for megabenthic and demersal fish fauna. The dashed line represents an (imaginary) horizontal reference line of one m width alongside the laser beams. Circles identify the three individuals/colonies of megabenthic fauna that were annotated in this video frame. Abbreviations: record inside (IN) and outside (OUT) of the horizontal reference line.


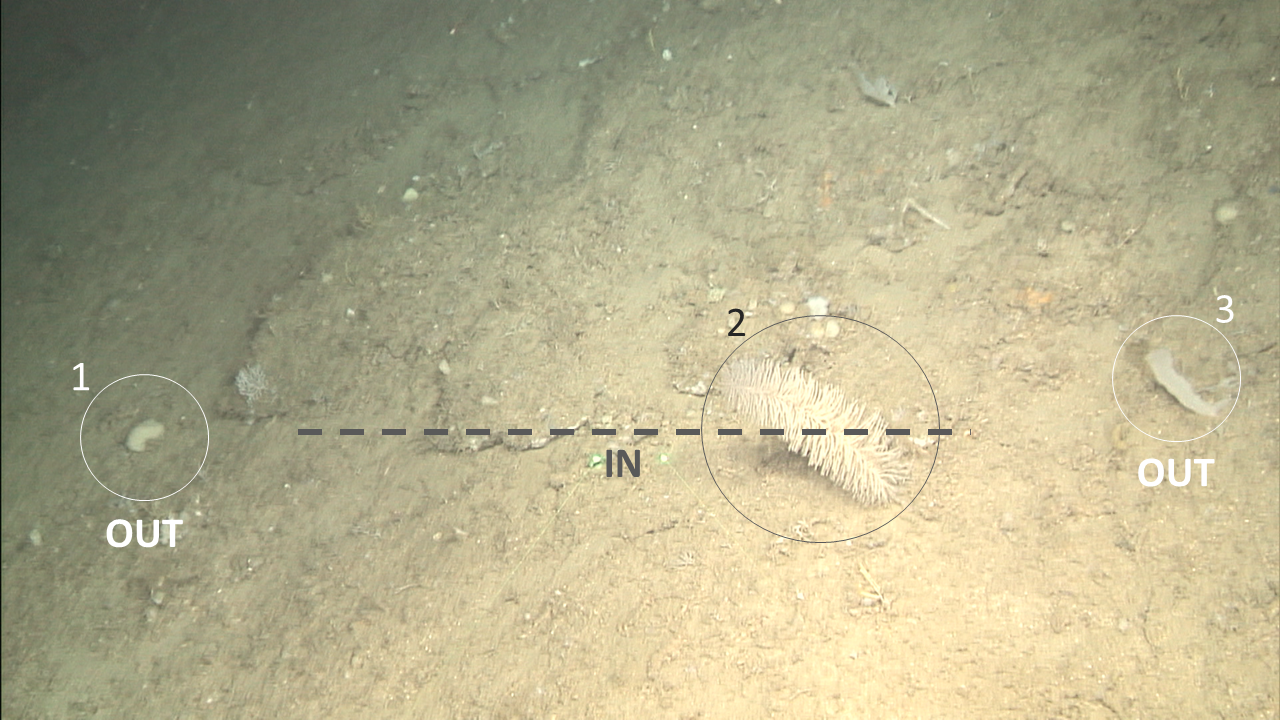


**Figure S2.** Faunistic composition of the video transects based on the main taxonomic groups. (a) Annotations made only within the 1 m width reference line (IN). (b) Annotations using the entire video frame (IN+OUT).

**a)**

**b)**

**
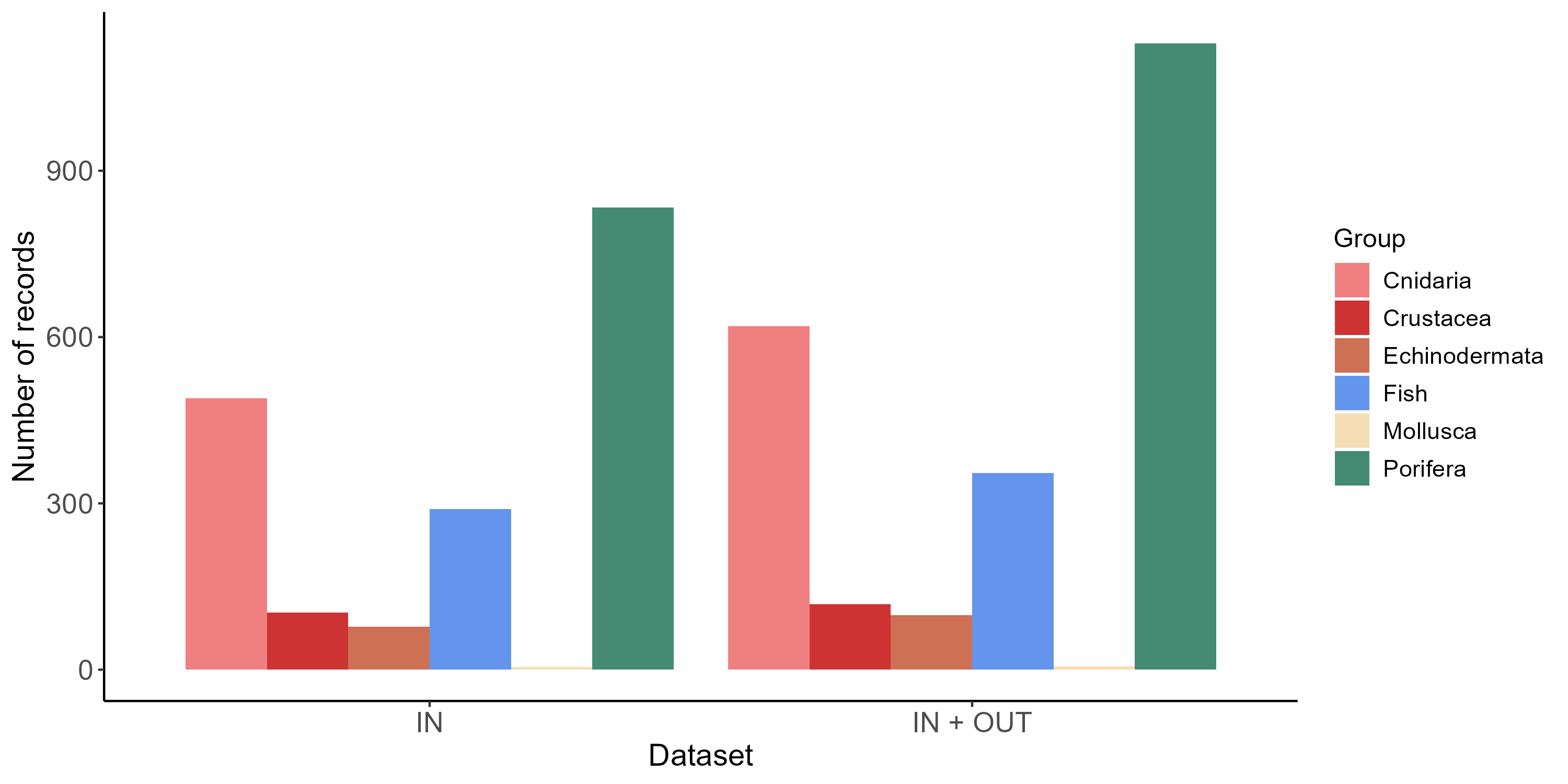
**

**Figure S3.** Assemblages identified at the the Seco de los Olivos Bank. a) Assemblage 1: *Muddy bottoms with cerianthids and sea pens. b)* Assemblage 2: *Soft bottoms dominated by Gadiculus argenteus*. c) Assemblage 3: *Mixed assemblage of small sponges and Callionymidae on soft bottoms. d)* Assemblage 4: *Hard bottoms with habitat-forming Porifera and Cnidaria. e)* Assemblage 5: *Soft bottoms dominated by Helicolenus dactylopterus*.


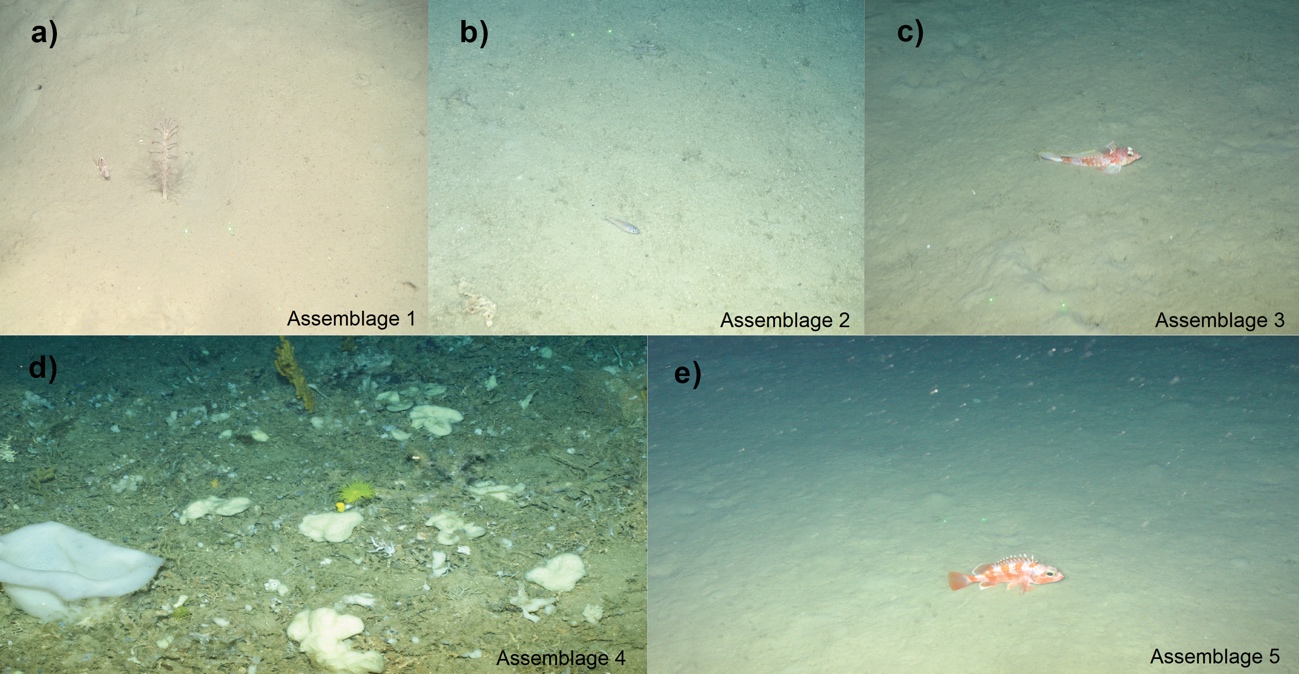


**Figure S4.** Examples of the indicators of anthropogenic activities observed at the Seco de los Olivos Bank, either defined as marine litter, remains of fishing gears and trawl marks. a) Cans in vicinity of juveniles of the fish *Helicolenus dactylopterus*, b) *H. dactylopterus* hidden inside a concrete structure from fishing gear remains, c) the sponge *Asconema setubalense* crossed by a fishing line, d) large accumulation of fishing lines and net remains entangled on octocorals, e) fishing line over a gorgonian colony and f) trawl mark on a muddy bottom.


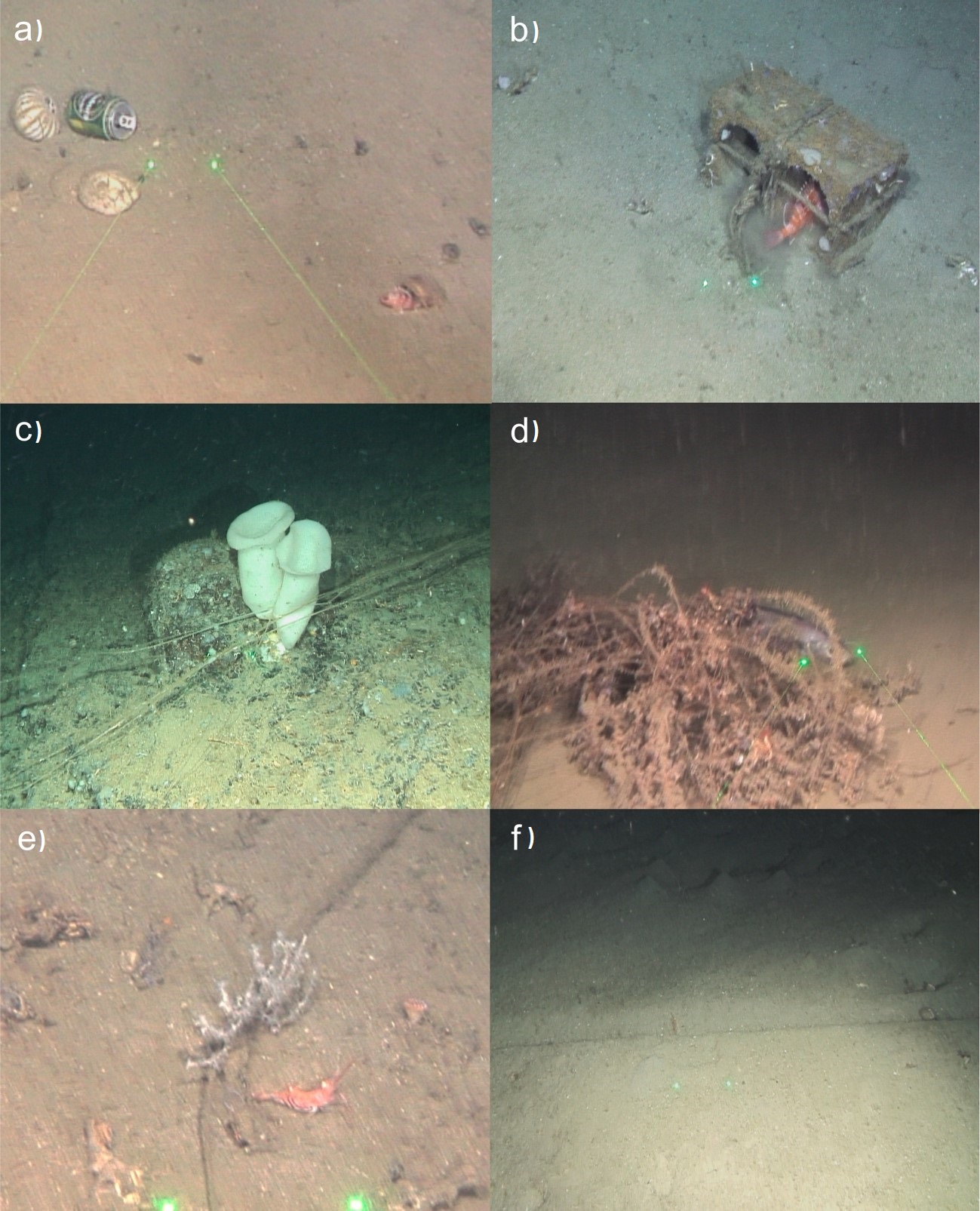


**Table S1.** List of the taxa identified at the Seco de los Olivos Bank, including ID number for the identification in the fauna catalogue (Cañedo-Apolaya et al., 2020), major taxonomic group, taxon name (i.e., open nomenclature to the best possible taxonomic identification), description (only for taxa not identified at the species level), observed depth range (minimum, maximum) in the present study and IUCN conservation status in 2023 (DD= data deficient, LC= least concerned, VU= vulnerable, NT= near threatened, EN= endangered) at Mediterranean (M), European (E) and global (G) level.

| **ID number** | **Group** | **Taxon** | **Description** | **Min depth (m)** | **Max depth (m)** | **IUCN status** |
| --- | --- | --- | --- | --- | --- | --- |
| 1 | Porifera | *Asconema setubalense* Kent, 1870 |  | 244 | 390 | - |
| 2 | Porifera | *Lycopodina hypogea* Vacelet & Boury-Esnault, 1996 |  | zzzz |  | - |
| 3 | Porifera | Suberitidae gen. indet. | Spherical and ~ 5 cm. This taxon includes *Aaptos aapto*s Schmidt, 1864 and other species. | 242 | 667 | - |
| 4 | Porifera | Tetillidae gen. Indet. | Ball-shaped and > 10 cm. This taxon resembles genus *Craniella*. | 240 | 245 | - |
| 5 | Porifera | Heteroscleromorpha fam. stet. | Fan-shaped sponges that may include *Phakellia robusta* Bowerbank, 1866, *Phakellia* sp. indet., *Pachastrella monilifera* Schmidt, 1868, and *Poecillastra* sp. indet. | 240 | 447 | - |
| 6 | Porifera | Porifera fam. inc. sp.1 | Compact structure without definable shape, usually color yellow. This taxon could include *Haliclona mucosa* Griessinger, 1971. | 239 | 449 | - |
| 7 | Porifera | Porifera fam. inc. sp.2 | Compact structure without definable shape, usually color white. | 240 | 443 | - |
| 8 | Porifera | Porifera fam. inc. sp.3 | Compact structure without definable shape. Usually color orange. | 239 | 365 | - |
| 9 | Porifera | Cladocroce spp. indet. | Fan-shaped and cup-shaped sponges with a peduncle. This taxon could inlcude *Cladocroce fibrosa* Topsent, 1890. | 274 | 667 | - |
| 10 | Porifera | Porifera fam. inc. sp.4 | Funnel shaped with a peduncle. Usually color white. | 240 | 336 | - |
| 11 | Porifera | Bubaridae gen. inc. | Funnel shaped. Usually, color light yellow. Possibly *Phakellia ventilabrum* Linnaeus, 1767 | 281 | 389 | - |
| 12 | Porifera | Heteroscleromorpha fam. indet. | Lollipop-shaped sponges with a large, circular to oval and apically compressed "head" and frequently, with a slender peduncle. This taxon may include *Rhizaxinella pyrifera* Delle Chiaje, 1828. | 245 | 630 | - |
| 13 | Porifera | Porifera fam. inc. sp.5 | Finger-like outgrowths from basal, as a single or divided. | 245 | 472 | - |
| 14 | Porifera | Hexactinellida fam. inc | Tube-shaped sponge, color white. This morphotype may include juveniles of *Asconema setubalense* and probably other glass sponges. | 243 | 379 | - |
| 15 | Porifera | Porifera fam. inc. 1 | All massive sponge specimens with an irregular shape that do not fit in the others categories | 241 | 449 | - |
| 16 | Porifera | Porifera fam. inc. 2 | All specimens that look like sponge with a peduncle that do not fit in the others categories. | 310 | 403 | - |
| 17 | Porifera | Porifera fam. inc. 3 | All unknown sponges with different shapes that do not fit in the others categories. | 248 | 677 | - |
| 18 | Cnidaria | *Kophobelemnon* *stelliferum* Müller, 1776 |  | 366 | 764 | - |
| 19 | Cnidaria | *Pennatula* cf. *Aculeata,* Linnaeus, 1758 |  | 525 | 774 | - |
| 20 | Cnidaria | *Funiculina quadrangularis* Pallas, 1766 |  | 246 | 550 | VU (M) |
| 21 | Cnidaria | *Callogorgia verticillata* Pallas, 1766 |  | 248 | 356 | NT (M) |
| 22 | Cnidaria | *Acanthogorgia* spp. indet. | Taxon includes *A. hirsuta* Gray, 1857 and *A. armata* Verrill, 1878 | 239 | 405 | - |
| 23 | Cnidaria | Malacalcyonacea spp. indet. | This taxon may include juveniles of *Acanthogorgia* spp. indet., *Viminella flagellum* Johnson, 1863 and *Bebryce mollis* Philippi, 1842. | 239 | 378 | - |
| 24 | Cnidaria | *Dendrophyllia cornigera* Lamarck, 1816 |  | 239 | 365 | EN (M) |
| 25 | Cnidaria | *Parantipathes larix* Esper, 1788 |  | 239 | 670 | NT (M) |
| 26 | Cnidaria | *Madrepora oculata* Linnaeus, 1758 |  | 239 | 332 | EN (M) |
| 27 | Cnidaria | *Desmophyllum pertusum* Linnaeus, 1758 | Previously known as *Lophelia pertusa*. | 240 | 329 | EN (M) |
| 28 | Cnidaria | *Savalia savaglia* Bertoloni, 1819 |  | 244 | 248 | NT (M) |
| 29 | Cnidaria | Ceriantharia fam. indet. | It may include several species. | 364 | 772 | - |
| 30 | Cnidaria | Actiniaria fam. indet. |  | - |  | - |
| 31 | Cnidaria | *Nicella granifera* Kölliker, 1865 |  | - |  | - |
| 32 | Cnidaria | *Muriceides lepida* Carpine & Grasshoff, 1975 |  | - |  | DD (M) |
| 33 | Cnidaria | Caryophylliidae gen. Indet. |  | - |  | - |
| 34 | Bryozoa | *Reteporella* sp. indet. |  | - |  | - |
| 35 | Annelida | *Bonellia* cf. v*iridis,* Rolando, 1822 |  | - | 627 | - |
| 36 | Annelida | *Filograna implexa* Berkeley, 1835 |  | - |  | - |
| 37 | Mollusca | *Eledone cirrhosa* Lamarck, 1798 |  | - | 242 | LC (G) |
| 38 | Mollusca | Neopycnodonte zibrowii |  | - | 672 | - |
| 39 | Mollusca | *Ranella olearium* Linnaeus, 1758 |  | 242 | 312 | - |
| 40 | Mollusca | Coleoidea sp. indet | Squid probably from Ommathrephidae family | - |  | - |
| 41 | Arthropoda:Crustacea | *Bathynectes* cf. *maravigna* |  | 381 | 403 | - |
| 42 | Arthropoda:Crustacea | *Geryon longipes* A. Milne-Edwards, 1882 |  | - |  | - |
| 43 | Arthropoda:Crustacea | *Munida* spp. indet. |  | 249 | 781 | - |
| 44 | Arthropoda:Crustacea | Paguridae spp. stet. | This taxon includes *Dardanus* spp. indet. and possibly other species. | 322 | 748 | - |
| 45 | Arthropoda:Crustacea | *Plesionika* spp. indet. |  | 233 | 657 | - |
| 46 | Echinodermata | Goniasteridae spp. indet. | This taxon includes *Peltaster placenta* Müller & Troschel, 1842 and similar starfish. | 328 | 409 | - |
| 47 | Echinodermata | *Hymenodiscus coronata* Sars, 1871 |  | 514 | 728 | - |
| 48 | Echinodermata | Ophiuridae gen. indet. |  | - |  | - |
| 49 | Echinodermata | *Ophiothrix* sp. indet. |  | - |  | - |
| 50 | Echinodermata | *Parastichopus* spp. indet. | It probably includes the species *P. tremulus* Gunnerus, 1767 and *P. regalis* Cuvier, 1817 | 249 | 793 | - |
| 51 | Echinodermata | *Mesothuria intestinalis* Ascanius, 1805 |  | 424 | 728 | LC (G) |
| 52 | Echinodermata | Holothuroidea gen. indet | It may include more than one species | 246 | 728 | - |
| 53 | Echinodermata | Echinidae gen. indet. | It may include more than one species | - | 308 | - |
| 54 | Echinodermata | Cidaridae spp. indet | This taxon includes *Cidaris cidaris* Linnaeus, 1758 and possibly *Stylocidaris affinis* Philippi, 1845. | 241 | 772 | - |
| 55 | Echinodermata | Crinoidea spp. indet. |  | - |  | - |
| 56 | Chordata:Vertebrata | *Scyliorhinus canicula* Linnaeus, 1758 |  | 353 | 340 | LC (M, E, G) |
| 57 | Chordata:Vertebrata | *Galeus* sp. indet. |  | 358 | 793 |  |
| 58 | Chordata:Vertebrata | *Coelorinchus caelorhincus* Risso, 1810 |  | 285 | 625 | LC (M, G) ; DD (E) |
| 59 | Chordata:Vertebrata | *Hymenocephalus italicus* Giglioli, 1884 |  | 248 | 615 | LC (M, E, G) |
| 60 | Chordata:Vertebrata | *Nezumia aequalis* Günther, 1878 |  | 500 | 771 | LC (M, E, G) |
| 61 | Chordata:Vertebrata | *Phycis blennoides* Brünnich, 1768 |  | 512 | 791 | LC (M) ; DD (E) |
| 62 | Chordata:Vertebrata | *Gadiculus argenteus* Guichenot, 1850 |  | 333 | 441 | LC (M, E) |
| 63 | Chordata:Vertebrata | *Micromesistius poutassou* Risso, 1827 |  | 290 | 300 | LC (M, E) |
| 64 | Chordata:Vertebrata | *Helicolenus dactylopterus* Delaroche, 1809 |  | 242 | 590 | LC (M, E, G) |
| 65 | Chordata:Vertebrata | *Scorpaena elongata* Cadenat, 1943 |  | 245 | 296 | LC (M, E, G) |
| 66 | Chordata:Vertebrata | *Trigla lyra* Linnaeus, 1758 |  | - | 284 | LC (M, G) ; DD (E) |
| 67 | Chordata:Vertebrata | *Hoplostethus mediterraneus* Cuvier, 1829 |  | 310 | 580 | LC (E, G) |
| 68 | Chordata:Vertebrata | *Conger conger* Linnaeus, 1758 |  | - | 261 | LC (E, G) |
| 69 | Chordata:Vertebrata | *Anthias anthias* Linnaeus, 1758 |  | 244 | 248 | LC (E, G) |
| 70 | Chordata:Vertebrata | *Capros aper* Linnaeus, 1758 |  | 243 | 385 | LC (M, E, G) |
| 71 | Chordata:Vertebrata | Callionymidae spp. indet. | This taxon may include *Synchiropus phaeton* Günther, 1861 and possibly other species. | 231 | 373 |  |
| 72 | Chordata:Vertebrata | *Pagellus bogaraveo*Brünnich, 1768 |  | 313 | 335 | LC (M) ; NT (E, G) |
| 73 | Chordata:Vertebrata | *Trachurus trachurus* Linnaeus, 1758 |  | 228 | 793 | LC (M, E) ; VU (G) |
| 74 | Chordata:Vertebrata | Paralepididae spp. indet. |  | 249 | 793 |  |
| 75 | Chordata:Vertebrata | Epigonidae spp. indet. |  | 239 | 793 |  |

**Table S2.** Characterization and similarities of the assemblages identified by SIMPER analysis, including average similarity, minimum and maximum depth, the individual contribution (Contri %) and the cumulative contribution (Cum %) of the most representative taxa and to the intragroup similarity.

| **Assemblage** | **Average similarity (%)** | **Min Depth (m)** | **Max Depth (m)** | **Taxa** | **Contri**  **(%)** | **Cum**  **(%)** |
| --- | --- | --- | --- | --- | --- | --- |
|  |  |  |  |  |  |  |
| As1 | 26.16 | 365.5 | 773.9 | Ceriantharia fam. indet. | 54.03 | 54.03 |
|  |  |  |  | *Kophobelemnon stelliferum* | 34.95 | 88.98 |
| As2 | 43.82 | 342.7 | 756.9 | *Gadiculus argenteus* | 77.18 | 77.18 |
|  |  |  |  | *Pagellus bogaraveo* | 22.82 | 100 |
| As3 | 35.35 | 230.6 | 628.5 | Heteroscleromorpha fam. indet. | 71.94 | 71.94 |
|  |  |  |  | Callionymidae spp. indet. | 23.54 | 95.47 |
| As4 | 21.06 | 239 | 672.1 | Porifera fam. inc. 1 | 23.71 | 23.71 |
|  |  |  |  | *Parantipathes larix* | 11.66 | 35.37 |
|  |  |  |  | Porifera fam. inc. sp. 1 | 9.66 | 45.03 |
|  |  |  |  | Heteroscleromorpha fam. stet | 9.15 | 54.18 |
|  |  |  |  | *Dendrophyllia cornigera* | 7.7 | 61.88 |
| As5 | 36.48 | 248.8 | 625.5 | *Helicolenus dactylopterus* | 79.89 | 79.89 |
|  |  |  |  | *Coelorinchus caelorinchus* | 15.7 | 95.58 |
